# Distribution and compartmental organization of GABAergic medium-sized spiny neurons in the mouse nucleus accumbens

**DOI:** 10.3389/fncir.2013.00022

**Published:** 2013-02-19

**Authors:** Giuseppe Gangarossa, Julie Espallergues, Alban de Kerchove d'Exaerde, Salah El Mestikawy, Charles R. Gerfen, Denis Hervé, Jean-Antoine Girault, Emmanuel Valjent

**Affiliations:** ^1^CNRS, UMR-5203, Institut de Génomique FonctionnelleMontpellier, France; ^2^Inserm, U661Montpellier, France; ^3^Universités de Montpellier 1 & 2, UMR-5203Montpellier, France; ^4^Laboratory of Neurophysiology, School of Medicine, Université Libre de Bruxelles, ULB Neuroscience InstituteBrussels, Belgium; ^5^CNRS, UMR-7224Paris, France; ^6^Inserm, U952Paris, France; ^7^Université Pierre et Marie Curie, UMR-7224Paris, France; ^8^Laboratory of Systems Neuroscience, National Institute of Mental HealthBethesda, MD, USA; ^9^Inserm, UMR-S 839Paris, France; ^10^Université Pierre et Marie Curie, UMR-S 839Paris, France; ^11^Institut du Fer á MoulinParis, France

**Keywords:** medium-sized spiny neurons, BAC transgenic, nucleus accumbens, dopamine, psychostimulant, ERK signaling, neural circuits

## Abstract

The nucleus accumbens (NAc) is a critical brain region involved in many reward-related behaviors. The NAc comprises major compartments the core and the shell, which encompass several subterritories. GABAergic medium-sized spiny neurons (MSNs) constitute the output neurons of the NAc core and shell. While the functional organization of the NAc core outputs resembles the one described for the dorsal striatum, a simple classification of the NAc shell neurons has been difficult to define due to the complexity of the compartmental segregation of cells. We used a variety of BAC transgenic mice expressing enhanced green fluorescence (EGFP) or the Cre-recombinase (Cre) under the control of the promoter of dopamine D1, D2, and D3 receptors and of adenosine A2a receptor to dissect the microanatomy of the NAc. Moreover, using various immunological markers we characterized in detail the distribution of MSNs in the mouse NAc. In addition, cell-type specific extracellular signal-regulated kinase (ERK) phosphorylation in the NAc subterritories was analyzed following acute administration of SKF81297 (a D1R-like agonist), quinpirole (a D2 receptors (D2R)-like agonist), apomorphine (a non-selective DA receptor agonist), raclopride (a D2R-like antagonist), and psychostimulant drugs, including cocaine and d-amphetamine. Each drug generated a unique topography and cell-type specific activation of ERK in the NAc. Our results show the existence of marked differences in the receptor expression pattern and functional activation of MSNs within the shell subterritories. This study emphasizes the anatomical and functional heterogeneity of the NAc, which will have to be considered in its further study.

## Introduction

Located in the rostrobasal forebrain, the nucleus accumbens (NAc) is a major brain area that processes incentive–reward responses associated with novel, hedonic, stressful, or aversive stimuli (Kalivas and Duffy, 1995; Reynolds and Berridge, 2002; Jensen et al., 2003; Nicola, 2007). Dysfunctions of this structure have been associated with prominent psychiatric disorders including obsessive-compulsive disorder, depression, and drug addiction (Nicola, 2007; Sesack and Grace, 2010).

Generally seen as an integral part of the striatal complex, it is, however, widely accepted that the NAc represents an independent entity that exhibits unique features compared with the dorsal striatum (Herkenham et al., 1984). Using a variety of immunohistochemical markers and tract-tracing approaches, earlier studies allowed distinguishing three major compartments in the NAc, the rostral pole, the core and the shell (Zaborszky et al., 1985; Zahm and Brog, 1992), but also multiple subterritories within these three compartments (Heimer et al., 1991; Zahm and Brog, 1992; Jongen-Relo et al., 1993, 1994; Groenewegen et al., 1999).

The NAc lacks glutamatergic neurons but is instead mostly composed of GABAergic medium-sized spiny neurons (MSNs), the remaining neurons being cholinergic and GABAergic interneurons (Meredith et al., 1993). The functional organization of the NAc core MSNs resembles that described for the dorsal striatum. Indeed, NAc core MSNs can be categorized into at least two different subgroups according to their projections sites. The MSNs projecting to the ventral tegmental area (VTA) express exclusively D1 receptors (D1R) resembling therefore the striatonigral MSNs (Gerfen et al., 1990; Schiffmann et al., 1991; Fink et al., 1992; Gerfen, 1992; Le Moine and Bloch, 1995). However, pallidal afferents from the NAc appear to differ from the striatopallidal MSNs of the dorsal striatum since NAc core MSNs innervating the ventral pallidum (VP) express D1R and D2 receptors (D2R)/adenosine A2a receptors (A2aR) (Lu et al., 1998). Due to the diversity of their output targets that include VTA, hypothalamus, VP, and brainstem, a simple division into direct and indirect pathways has been even more difficult to define for the NAc shell MSNs (Voorn et al., 1989; Zahm and Brog, 1992; Van Dongen et al., 2008; Humphries and Prescott, 2010; Sesack and Grace, 2010). In addition, a higher proportion of MSNs in the shell than in the rest of the striatum appears to co-express D1R and D2R, suggesting that the segregation in two distinct populations is far from being complete (Le Moine and Bloch, 1996; Bertran-Gonzalez et al., 2008, 2010; Matamales et al., 2009; Durieux et al., 2011). Moreover, whether D1R- and D2R-expressing MSNs are randomly distributed or exhibit inhomogeneous distribution patterns in the different subterritories of the NAc shell remains to be established.

To address these issues, we took advantage of BAC transgenic mice expressing enhanced green fluorescent protein (EGFP) under the control of the promoter of D1R, *Drd1a-EGFP*, and D2R, *Drd2-EGFP* (Gong et al., 2003; Valjent et al., 2009). Moreover, the dopamine D3 receptor (D3R) being highly expressed in the NAc, we also analyzed GFP expression in *Drd3-Cre* crossed with the *Rosa26:loxP* reporter mouse line. Using a variety of immunological markers we characterized in detail the microanatomical distribution of D1R- and D2R-expressing MSNs in the mouse NAc. We also provide evidence that dopaminergic agonists and psychostimulant drugs induce specific and topographical patterns of extracellular signal-regulated kinase (ERK) activation that are closely associated with specific NAc shell subterritories.

## Materials and methods

### Animals

*Drd2-EGFP* (*n* = 29, Swiss-Webster background, founder *S118*), *Drd2-Cre* (*n* = 4, C57/Bl6J background, founder *ER44*), *Drd1a-EGFP* (*n* = 4, Swiss-Webster background, founder *X60*), *Drd3-Cre* (*n* = 2, C57/Bl6J background, founder *KI196*), and *Adora2a-Cre* (Durieux et al., 2009) (*n* = 3, C57/Bl6J background) BAC transgenic mice were used in this study. BAC-EGFP and BAC-Cre mice were originally generated by GENSAT (Gene Expression Nervous System Atlas) at the Rockefeller University (New York, NY) (Gong et al., 2003) except the *Adora2a-Cre* (Durieux et al., 2009). *Adora2a-Cre* mice were used to identify striatopallidal neurons. Indeed in the striatum, these mice expressed the Cre recombinase selectively in striatopallidal neurons but not in other striatal populations (striatonigral MSNs, GABA, and cholinergic interneurons) or in the presynatic DA neurons (Durieux et al., 2009). *Rosa26:loxP* (Srinivas et al., 2001) and ***R****26R* ***C****AG-boosted* ***E****GFP:LoxP* (*RCE:LoxP)* (Miyoshi et al., 2010) mice were used as reporter to compare the patterns of expression in different mouse lines. Male 8–10 week-old mice were used and maintained in a 12 h light/dark cycle, in stable conditions of temperature (22°C) and humidity (60%), with food and water *ad libitum*. For the pharmacological studies only *Drd2-EGFP* heterozygous mice were used. All experiments were in accordance with the guidelines of the French Agriculture and Forestry Ministry for handling animals (C34-172-13).

### Drugs and treatment

SKF81297 (5.0 mg/kg, i.p.), quinpirole (1.0 mg/kg, i.p.), apomorphine (3.0 mg/kg, s.c.), and raclopride (0.3 mg/kg, i.p.) were purchased from Tocris and dissolved in 0.9% (w/v) NaCl (saline). Cocaine (15 mg/kg, i.p.) and d-amphetamine (10 mg/kg, i.p.) were purchased from Sigma Aldrich and dissolved in 0.9% (w/v) NaCl (saline). Mice were habituated to handling and saline injection three consecutive days before the experiment. Drugs were administrated on day 4. All the mice were injected in the home cage and perfused 15 min after injection.

### 6-OHDA lesion

*Drd2-EGFP* mice were anaesthetized with a mixture of ketamine (Imalgene 500, 50 mg/ml, Merial), 0.9% NaCl solution (weight/vol), and xylazine (Rompun 2%, 20 mg/ml, Bayer) (2:2:1, i.p., 0.1 ml/30 g) and mounted on a stereotaxic apparatus. The surface of the skull was exposed and a hole was drilled at the appropriate coordinates. A cannula connected to a Hamilton 0.5 μl microsyringe was stereotaxically lowered to the VTA. The following coordinates were used: *AP* = −3.16, *L* = −0.55, and *V* = −4.5 (Franklin and Paxinos, 2007). A volume of 0.25 μl of 6-OHDA^*^HCl (3 μg/μl of free base, dissolved in ascorbic acid 0.02%) was unilaterally injected at a rate of 0.05 μl/min. The intra VTA microinjection of 6-OHDA was preceded (30 min) by administration of desipramine (20 mg/kg, i.p.) to avoid degeneration of noradrenergic fibers. Following injection the cannula was left in place for another 4 min before retraction. Mice were allowed to recover for a period of two 2 weeks before experiments.

### Tissue preparation and immunofluorescence

Mice were rapidly anaesthetized with pentobarbital (500 mg/kg, i.p., Sanofi-Aventis, France) and transcardially perfused with 4% (weight/vol.) paraformaldehyde in 0.1 M sodium phosphate buffer (pH 7.5). Brains were post-fixed overnight in the same solution and stored at 4°C. Thirty μm-thick sections were cut with a vibratome (Leica, France) and stored at −20°C in a solution containing 30% (vol/vol) ethylene glycol, 30% (vol/vol) glycerol, and 0.1 M sodium phosphate buffer, until they were processed for immunofluorescence. Sections were processed as follows: Day 1: free-floating sections were rinsed in Tris-buffered saline (TBS; 0.25 M Tris and 0.5 M NaCl, pH 7.5), incubated for 5 min in TBS containing 3% H_2_O_2_ and 10% methanol, and then rinsed three times for 10 min each in TBS. After 15 min incubation in 0.2% Triton X-100 in TBS, sections were rinsed three times in TBS again. Finally, they were incubated overnight at 4°C with the different primary antibodies. For detection of phosphorylated proteins, 50 mM NaF was included in all buffers and incubation solutions. Slices were then incubated overnight or 72 h at 4°C with the following primary antibodies: chicken, mouse and rabbit anti-GFP (1:500 and 1:1000, respectively, Invitrogen), rabbit anti-vesicular glutamate transporter 1 (VGluT1) or anti-VGluT2 (1:1000), rabbit anti-calretinin (1:1000, Swant), mouse and rabbit anti-tyrosine hydroxylase (TH) (1:1000, Millipore), rat anti-dopamine transporter (DAT) (1:1000, Millipore), mouse anti-DARPP-32 (1:1000 gift from P. Greengard), rabbit against diphospho-Thr-202/Tyr-204-ERK1/2 (1:400, Cell Signaling Technology). Sections were rinsed three times for 10 min in TBS and incubated for 45 min with goat Cy2-, Cy3-, and Cy5-coupled (1:500, Jackson Lab) and/or goat A488 (1:500, Invitrogen). Sections were rinsed for 10 min twice in TBS and twice in TB (0.25 M Tris) before mounting in 1,4-diazabicyclo-[2.2.2]-octane (DABCO, Sigma-Aldrich).

Confocal microscopy and image analysis were carried out at the Montpellier RIO Imaging Facility. All images covering the entire NAc were single confocal sections, acquired using sequential laser scanning confocal microscopy (Zeiss LSM780) and stitched together as a single image. Double-labeled images from each region of interest were also single confocal sections obtained using sequential laser scanning confocal microscopy (Zeiss LSM510 META and Zeiss LSM780). Photomicrographs were obtained with the following band-pass and long-pass filter setting: GFP/Cy2 (band pass filter: 505–530), Cy3 (band pass filter: 560–615), and Cy5 (long-pass filter 650). GFP-labeled neurons were pseudocolored cyan or green and other immunoreactive markers were pseudocolored red or magenta. From the overlap of cyan and red or green and magenta, double-labeled neurons appeared white. Images used for quantification were all single confocal sections. The objectives and the pinhole setting (1 airy unit) remain unchanged during the acquisition of a series for all images. The thickness of the optical section is ~1.6 μm with a 20× objective and ~6 μm with a 10× objective. P-ERK-positive cells were quantified in zones or regions of the same area (630 × 630 μm or 1273 × 1273 μm) in every shell subterritories delineated in each slice by TH immunoreactivity (Tables A1, A2). A similar analysis was performed to evaluate the percentage of GFP-positive cells expressing DARPP-32 in the different BAC transgenic mice used. Quantification of immunoreactive cells was performed using the cell counter plugin of the ImageJ software taking as standard reference a fixed threshold of fluorescence.

### Delineation of NAc core and NAc shell subterritories

Coronal sections, in which the core-shell boundary was clearly visible, between bregma 1.34 and 0.98 mm, were selected for analysis (Franklin and Paxinos, 2007). The NAc core and shell delineation was done based upon calbindin-D 28 kDa (strongly enriched in the core compared to the shell) and calretinin immunostainings (strongly enriched in the shell compared to the core). In addition to the differential expression of GFP in the different mouse lines used, the delineation of the substructures of the NAc shell was based upon a combination of markers including TH, DAT, VGluT1, VGluT2, and calretinin immunoreactivities. The striatal-enriched phosphoprotein DARPP-32 was used to identify MSNs (Ouimet et al., 1984).

### Statistical analysis

Data were analyzed using one-way ANOVA, where treatment was the independent variable, followed by Dunnett's *post hoc* test for specific comparisons. Differences were considered significant when *p* < 0.05.

## Results

### Topographical organization of D1R- and D2R-expressing MSNs in the NAc

**Figure 1:**
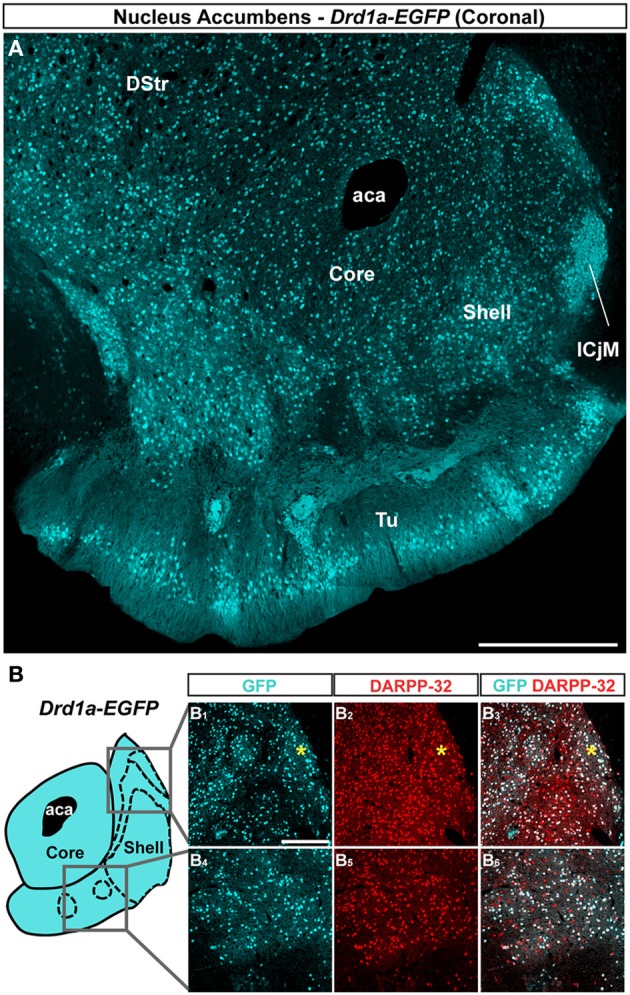
**Topographical distribution of GFP immunofluorescence in the NAc of *Drd1a-EGFP* mice. (A)** Distribution of GFP in the NAc of *Drd1a-EGFP* BAC transgenic mice. Single confocal sections were stitched together as a single image. Scale bar: 500 μm. DStr, dorsal striatum; aca, anterior commissure; ICjM, islands of Calleja major; Tu, olfactory tubercles. **(B)** Schematic illustration of the different accumbal subterritories analyzed (see Figure A1). Cyan in the diagram represents distribution of GFP-expressing cells. A zone homogenously cyan means the GFP is homogenously distributed. Note that D1R-containing MSNs show a relative homogeneous distribution. Single scan confocal images stained for GFP (cyan) and DARPP-32 (red), a marker of MSNs in the dorsal caudomedial (GFP, **B**_**1**_; DARPP-32, **B**_**2**_; merge, **B**_**3**_) and ventral (GFP, **B**_**4**_; DARPP-32, **B**_**5**_; merge, **B**_**6**_) part of the shell of *Drd1a-EGFP* mice. Images shown are representative of all *Drd1a-EGFP* BAC transgenic mice analyzed (*n* = 4). Yellow asterisk identifies the D2R-expressing MSNs-poor zone. Scale bar: 200 μm.

**Figure 2:**
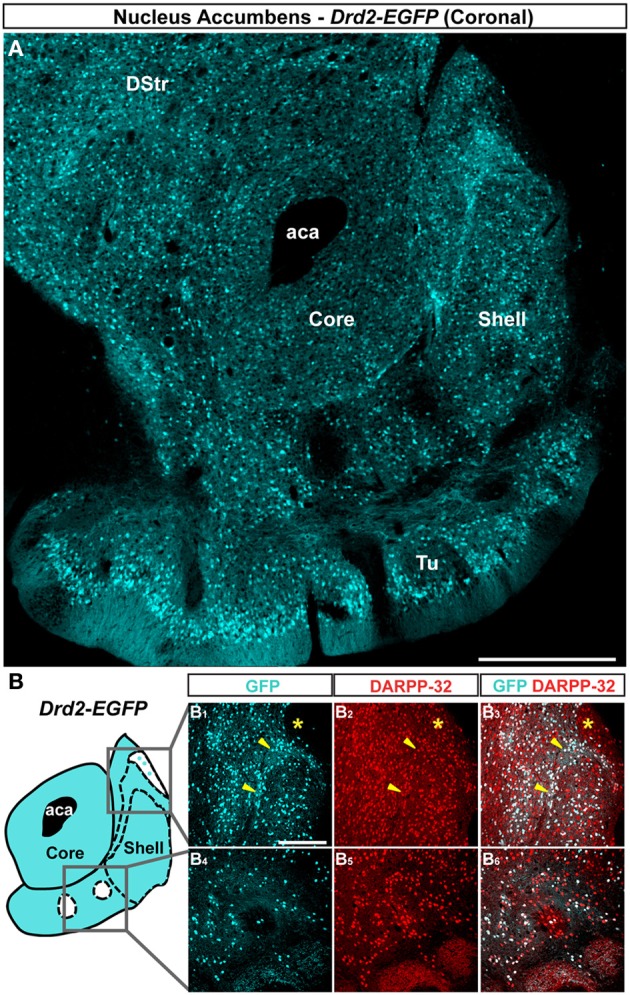
**Topographic distribution of GFP immunofluorescence in the NAc of *Drd2-EGFP* mice. (A)** Distribution of GFP in the NAc of *Drd2-EGFP* BAC transgenic mice. Single confocal sections were stitched together as a single image. Scale bar: 500 μm. DStr, dorsal striatum; aca, anterior commissure; Tu, olfactory tubercles. **(B)** Schematic illustration of the different accumbal subterritories analyzed (see Figure A1). Cyan in the diagram represents distribution of GFP-expressing cells. A zone homogenously cyan means the GFP is homogenously distributed. White means lack of GFP expression throughout the zone and dots indicate that few GFP cells are detected throughout the zone. Note that D2R-expressing MSNs exhibit inhomogeneous distributions in the NAc shell. In the caudomedial NAc shell (1) a bundle-shaped area and (2) a D2R-expressing MSNs-poor zones (white) in the upper part of the caudomedial shell (cyan) can be visualized. In the ventral shell, two zones lacking D2R-expressing MSNs (white) can be delineated. Single scan confocal images stained for GFP (cyan) and DARPP-32 (red), a marker of MSNs, in the dorsal caudomedial (GFP, **B**_**1**_; DARPP-32, **B**_**2**_; merge, **B**_**3**_) and ventral (GFP, **B**_**4**_; DARPP-32, **B**_**5**_; merge, **B**_**6**_) part of the shell of *Drd2-EGFP* mice. Images shown are representative of all *Drd2-EGFP* BAC transgenic mice analyzed (*n* = 5). Yellow asterisk identifies the D2R-expressing MSNs-poor zone. Yellow arrowheads identify the bundle-shaped area. Scale bar: 200 μm.

The analysis of GFP fluorescence in *Drd1a-EGFP* and *Drd2-EGFP* mice showed a relatively uniform appearance in the NAc core (Figures 1A, 2A). As previously observed, D1R- and D2R-expressing MSNs were homogeneously distributed in the NAc core and all the GFP-positive neurons were DARPP-32-immunoreactive MSNs with the exception of ChAT interneurons identified in *Drd2-EGFP* mice (data not shown; Bertran-Gonzalez et al., 2008; Matamales et al., 2009). Altogether, these observations revealed that D1R- and D2R-MSNs in the NAc core lack anatomical segregation, displaying instead a mixed organization that resembles that of the dorsal striatum.

**Figure A1:**
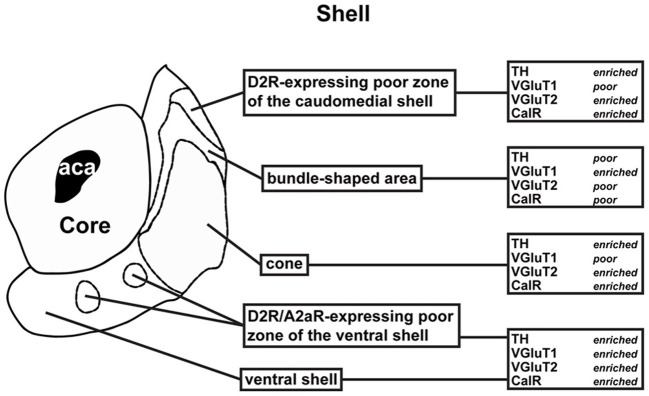
**Schematic representation of the subterritories of the caudomedial NAc shell analyzed.** Each region was defined based on a review of previous anatomical and neurochemical studies. See text for more detailed description.

On coronal sections used in this study, the core is surrounded on its medial, ventral and lateral sides by the shell (Figures 1A, 2A). As in the NAc core, no apparent organization was observed in the NAc shell in *Drd1a-EGFP* mice (Figure 1A). All D1R-expressing neurons were DARPP-32-positive in the medial, ventral and lateral shell (Figure 1B and data not shown) confirming they are all MSNs (Bertran-Gonzalez et al., 2008). In contrast, a complex and inhomogeneous distribution of D2R-expressing MSNs was observed in the shell in *Drd2-EGFP* mice (Figures 2, A1). The heterogeneous distribution of D2R-expressing neurons was particularly evident in the medial and ventral shell (Figure 2A). Thus, in the dorsal caudomedial part of the shell, several subterritories have been identified (Figure A1): (1) the “cone” region (Todtenkopf and Stellar, 2000), (2) a bundle-shaped area also termed corridors (Seifert et al., 1998), and (3) a D2R-expressing MSNs-poor zone in the upper part of caudomedial shell (Figures 2A,B,A1). In the ventral shell, D2R-lacking MSNs areas expressing DARPP-32-positive MSNs were identified (Figures 2A,B). A similar distribution was observed in *Drd2-Cre* mice crossed with the *RCE:LoxP* reporter line (Miyoshi et al., 2010) (data not shown).

### Topographical organization of A2aR-expressing MSNs in the NAc

**Figure 3:**
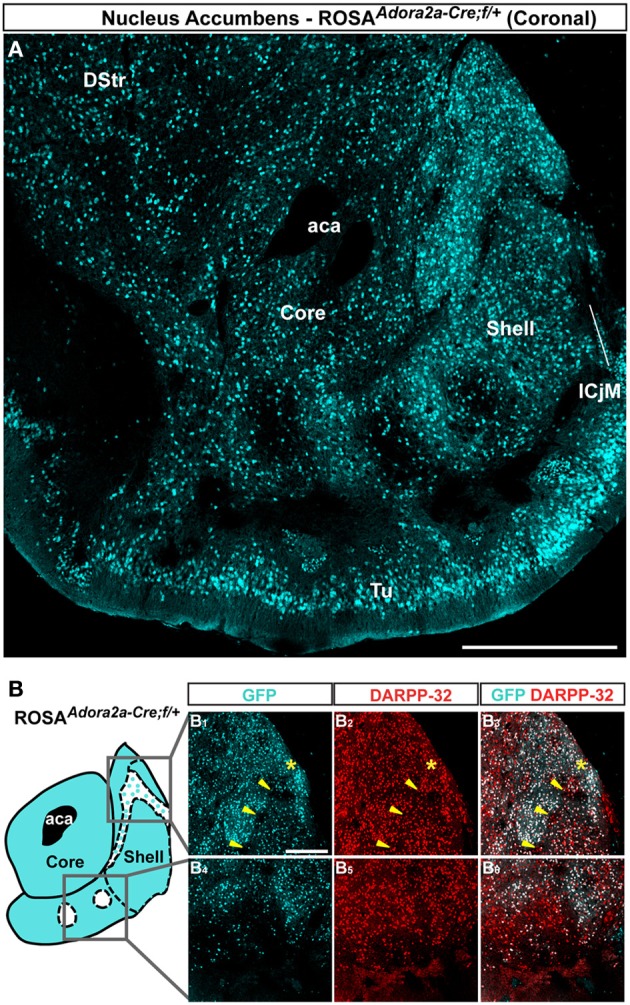
**Topographic distribution of GFP immunofluorescence in the NAc of *Adora2a-Cre* mice crossed with the *Rosa26:loxP* reporter line. (A)** Single confocal sections were stitched together as a single image. Scale bar: 500 μm. DStr, dorsal striatum; aca, anterior commissure; ICjM, islands of Calleja major; Tu, olfactory tubercles. **(B)** Schematic illustration of the accumbal subterritories analyzed (see Figure A1). Cyan in the diagram represents distribution of GFP-expressing cells. A zone homogenously cyan means the GFP is homogenously distributed. White means lack of GFP expression throughout the zone and dots indicate that few GFP cells are detected throughout the zone. Single scan confocal images showing double stained for GFP (cyan) and DARPP-32 (red) in the dorsal caudomedial (GFP, **B**_**1**_; DARPP-32, **B**_**2**_; merge, **B**_**3**_) and ventral (GFP, **B**_**4**_; DARPP-32, **B**_**5**_; merge, **B**_**6**_) part of the shell of *Adora2a-Cre* mice. Images shown are representative of all *Adora2a-Cre* BAC transgenic mice analyzed (*n* = 3). Yellow asterisk identifies the D2R-expressing MSNs-poor zone. Yellow arrowheads identify the bundle-shaped area. Scale bar: 200 μm.

Striatopallidal MSNs of the dorsal striatum express D2R and A2aR (Gerfen et al., 1990; Schiffmann et al., 1991; Fink et al., 1992; Le Moine and Bloch, 1995). We therefore analyzed the distribution of A2aR-expressing neurons in the NAc using *Adora2a-Cre* mice (Durieux et al., 2009) crossed with the *Rosa26:loxP* reporter line. In the NAc core, A2aR-expressing MSNs were homogeneously distributed (Figure 3A). As observed in *Drd2-EGFP* mice, a heterogeneous distribution of A2aR-expressing neurons was particularly evident in the medial and ventral shell (Figure 3A). Thus, GFP-positive cells that co-stained with DARPP-32 were detected in the bundle-shaped area as well as in the D2R-expressing MSNs-poor zone in the upper part of the caudomedial shell identified in *Drd2-EGFP* mice (Figure 3B). In the ventral shell, as observed in *Drd2-EGFP* mice, A2aR-lacking MSNs areas expressing DARPP-32-positive MSNs were identified (Figures 3A,B).

### Topographical organization of D3R-expressing MSNs in the NAc

**Figure 4:**
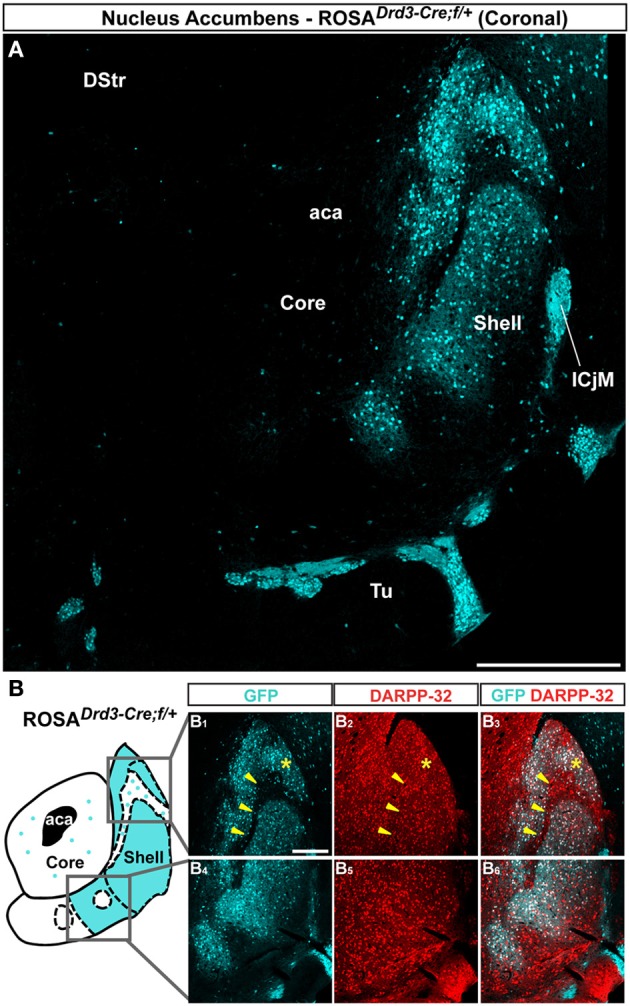
**Topographic distribution of GFP immunofluorescence in the NAc of *Drd3-Cre* mice crossed with the *Rosa26:loxP* reporter line. (A)** Single confocal sections were stitched together as a single image. Scale bar: 500 μm. DStr, dorsal striatum; aca, anterior commissure; ICjM, islands of Calleja major; Tu, olfactory tubercles. Note that GFP-positive cells are restricted to the caudomedial part of the NAc shell. Only sparse GFP-expressing cells were detected in the NAc core. **(B)** Schematic illustration of the accumbal subterritories analyzed (see Figure A1). Cyan in the diagram represents the distribution of GFP-expressing cells. A zone homogenously cyan means the GFP is homogenously distributed. White means lack of GFP expression throughout the zone and dots indicate that few GFP cells are detected throughout the zone. Single scan confocal images showing double stained for GFP (cyan) and DARPP-32 (red) in the dorsal caudomedial (GFP, **B_1_**; DARPP-32, **B_2_**; merge, **B_3_**) and ventral (GFP, **B**_**4**_; DARPP-32, **B**_**5**_; merge, **B**_**6**_) part of the shell of *Drd3-Cre* mice. Images shown are representative of all *Drd3-Cre* BAC transgenic mice analyzed (*n* = 2). Yellow asterisk identifies the D2R-expressing MSNs-poor zone. Yellow arrowheads identify the bundle-shaped area. Scale bar: 200 μm.

Unlike the dorsal part of the striatum, the NAc appears to be the area where the D3R is expressed at the highest level (Sokoloff et al., 1990; Bouthenet et al., 1991; Diaz et al., 1995; Le Moine and Bloch, 1996). We therefore investigated the distribution of D3R-expressing cells in the NAc by assessing the distribution of GFP-positive neurons in *Drd3-Cre* mice (http://www.gensat.org/cre.jsp) crossed with the *Rosa26:loxP* reporter line (Srinivas et al., 2001). Only few scattered GFP-positive cells that co-stained with DARPP-32 were detected in the NAc core of *Drd3-Cre* mice (Figure 4A). GFP-immunoreactive cells were absent in the lateral part of the shell and the lateral half of the ventral part (Figures 4A,B). D3R-expressing neurons were heterogeneously distributed in the medial part of the shell and in the medial half of the ventral part of the NAc shell (Figure 4A). Thus, the highest density of D3R-expressing cells was confined to the caudomedial shell (Figures 4A,B) while only few and scattered GFP-immunoreactive neurons were observed in the bundle-shaped area (Figures 4A,B). Double-immunofluorescence revealed that the majority of D3R-positive cells were MSNs since they co-localized with DARPP-32 (Figure 4B).

### Characterization of GFP-expressing neurons in the caudomedial part of the NAc shell

The bundle-shaped area and the D2R-expressing MSNs-poor zone of the upper part of the caudomedial shell displayed the most heterogeneous distribution pattern of MSNs in the mouse NAc. In an attempt to better characterize the accumbal circuitry in relationship to D1R-, D2R-, A2aR-, and D3R-expressing MSNs, we identified DARPP-32-positive MSNs in *Drd1a-EGFP*, *Drd2-EGFP*, *Adora2a-Cre*, and *Drd3-Cre* mice in these two subterritories and calculated the percentage of DARPP-32-immunostained neurons expressing GFP in each line.

**Figure 5:**
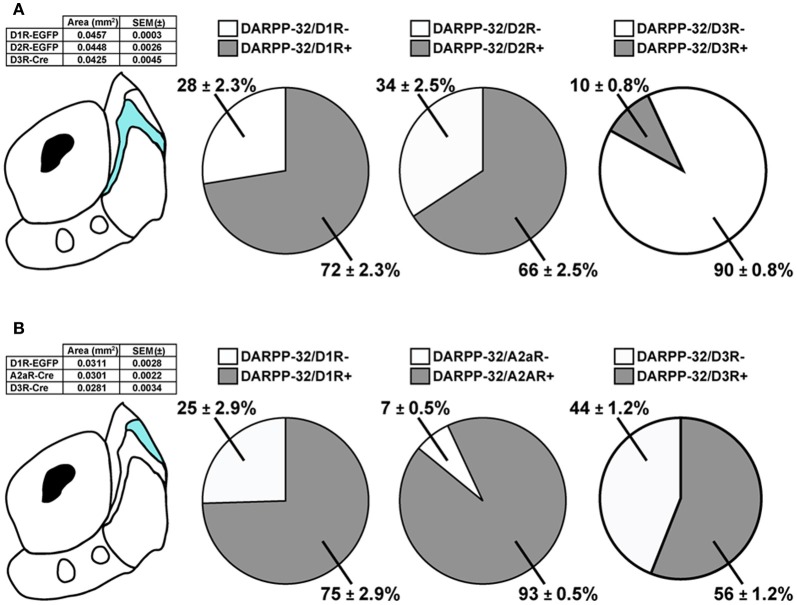
**Expression of D1R-, D2R-, A2aR-, and D3R-containing MSNs in subterritories of the caudomedial NAc shell. (A)** Percentage of DARPP-32-positive neurons expressing GFP in *Drd1a-EGFP*, *Drd2-EGFP*, and *Drd3-Cre* mice in bundle-shaped area of the caudomedial NAc shell. Quantifications were obtained from several images. Total number of MSNs counted: *n* = 950 MSNs in *Drd1a-EGFP* (eight hemispheres from four mice), *n* = 898 MSNs in *Drd2-EGFP* (eight hemispheres from four mice), and *n* = 629 MSNs in *Drd3-Cre* (eight hemispheres from two mice). The table summarizes the surface area analyzed for each mouse line. **(B)** Percentage of DARPP-32-positive neurons expressing GFP in *Drd1a-EGFP*, *Adora2a-Cre*, and *Drd3-Cre* mice in the D2R-expressing MSNs-poor zone of the caudomedial NAc shell. Quantifications were obtained from several images. Total number of MSNs counted: *n* = 329 MSNs in *Drd1a-EGFP* (six hemispheres from three mice), *n* = 349 MSNs in *Adora2-Cre* (six hemispheres from three mice), and *n* = 365 MSNs in *Drd3-Cre* (eight hemispheres from two mice). The table summarizes the surface area analyzed for each mouse line.

In the bundle-shaped area, 72 ± 2% and 66 ± 3% of the DARPP-32 immunoreactive neurons were GFP-positive in *Drd1a-EGFP* and *Drd2-EGFP* mice, respectively (Figure 5A). Although these numbers were obtained from different mice, the proportion of MSNs expressing D1R, D2R, or both was therefore roughly estimated from these data by adding the percentage of DARPP-32-positive neurons, which were GFP-positive in *Drd1a-EGFP* and *Drd2-EGFP* mice. This estimation is based on the assumption that every MSN express either D1R, D2R, or both, as previously shown (Matamales et al., 2009). The summed percentages obtained in *Drd1a-EGFP* and *Drd2-EGFP* mice exceeding 100% were taken as an indication of co-expression. This estimation revealed a high degree of D1R/D2R co-localization (38%) (72% of DARPP-32/D1R + 66% of DARPP-32/D2R = 138%) in this NAc shell subterritory while 34% (72% of DARPP-32/D1R—38% of DARPP-32/D2R/D1R = 34%) and 28% (66% of DARPP-32/D2R—38% of DARPP-32/D2R/D1R = 28%) of MSNs could express only D1R and D2R, respectively (Figure 5A). Moreover, we found that only a small proportion of the MSNs (~10%) located in this area expressed D3R (Figure 5A).

We performed the same analysis in the D2R-expressing MSNs-poor zone in the upper part of the caudomedial shell (Figure 5B). In this subregion, 75% of DARPP-32 immunoreactive neurons were found to be the GFP-positive in *Drd1a-EGFP*. However, the almost complete absence of D2R-expressing MSNs (~10%) raised the intriguing hypothesis that this subterritory is composed almost exclusively of D1R-containing MSNs. In the dorsal striatum D2R-containing MSNs co-express the adenosine A2aR (Schiffmann et al., 1991). We therefore analyzed the expression of GFP in *Adora2-Cre* mice (Durieux et al., 2009) crossed with the *Rosa26:loxP* reporter line (Srinivas et al., 2001). Surprisingly, we found that in the D2R-expressing MSNs-poor zone, 93% of DARPP-32-positive neurons were GFP-immunoreactive in *Adora2a-Cre* mice (Figure 5B). Using the same approach described above and if we assume that all MSNs of this subterritory express either D1R or A2aR or both, it can be estimated that 68% co-express both receptors (75% of DARPP-32/D1R + 93% of DARPP-32/A2aR = 168%), 7% (75% of DARPP-32/D1R—68% of DARPP-32/D1R/A2aR = 7%) of the MSNs express only D1R and 25% (93% of DARPP-32/A2aR—68% of DARPP-32/D1R/A2aR = 25%) express only A2aR. In addition, we also found that 56% of the DARPP-32-positive cells contained D3R (Figure 5B). However, all these calculations represent lower limits and should be taken with caution since it is not known whether some MSNs do not express either of these receptors.

### Immunochemical characterization of GFP-expressing neurons in the caudomedial part of the NAc shell

**Figure 6:**
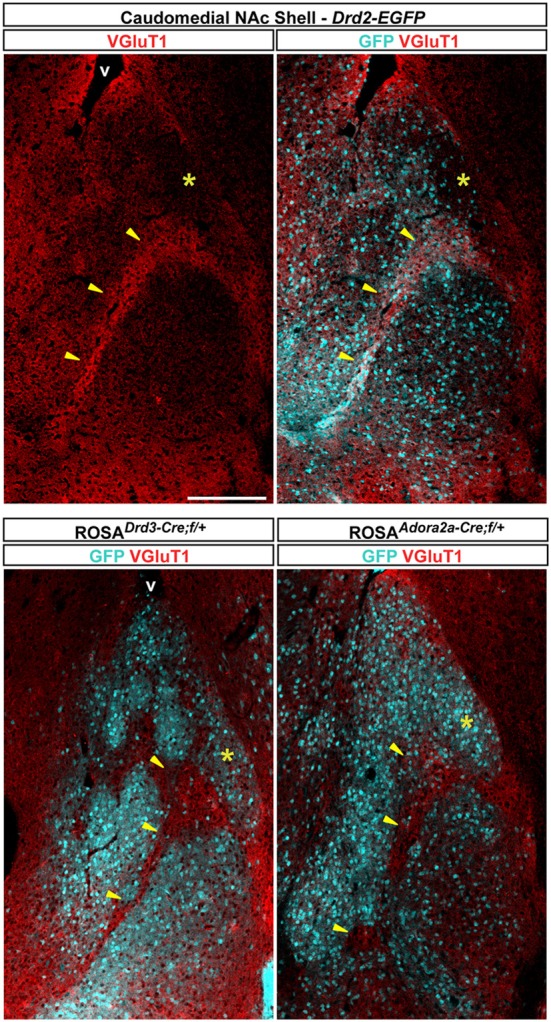
**Distribution pattern of vesicular transporter 1 (VGluT1) in the caudomedial NAc shell.** Distribution of VGluT1 (red) and GFP (cyan) immunofluorescence in the NAc of *Drd2-EGFP*, *Drd3-Cre/Rosa26:loxP* and *Adora2a-Cre/Rosa26:loxP* double transgenic mice. Images are single confocal sections. Yellow asterisk indicates the D2R-expressing MSNs-poor zone. Yellow arrowheads indicate the bundle-shaped area. Scale bar: 250 μm. v, ventricles.

**Figure 7:**
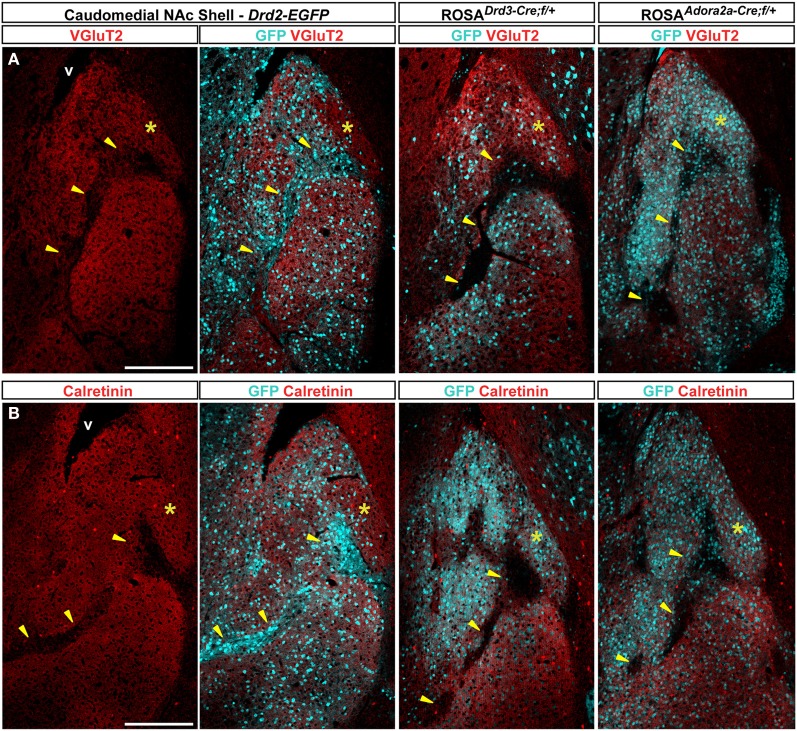
**Distribution patterns of vesicular transporter 2 (VGluT2) and Calretinin in the caudomedial NAc shell.** Distribution of VGluT2 (red) **(A)** or calretinin (red) **(B)** and GFP (cyan) fluorescence in the NAc of *Drd2-EGFP*, *Drd3-Cre/Rosa26:loxP* and *Adora2a-Cre/Rosa26:loxP* double transgenic mice. Images are single confocal sections. Yellow asterisk indicates the D2R-expressing MSNs-poor zone. Yellow arrowheads indicate the bundle-shaped area. Scale bar: 250 μm. v, ventricles.

**Figure 8:**
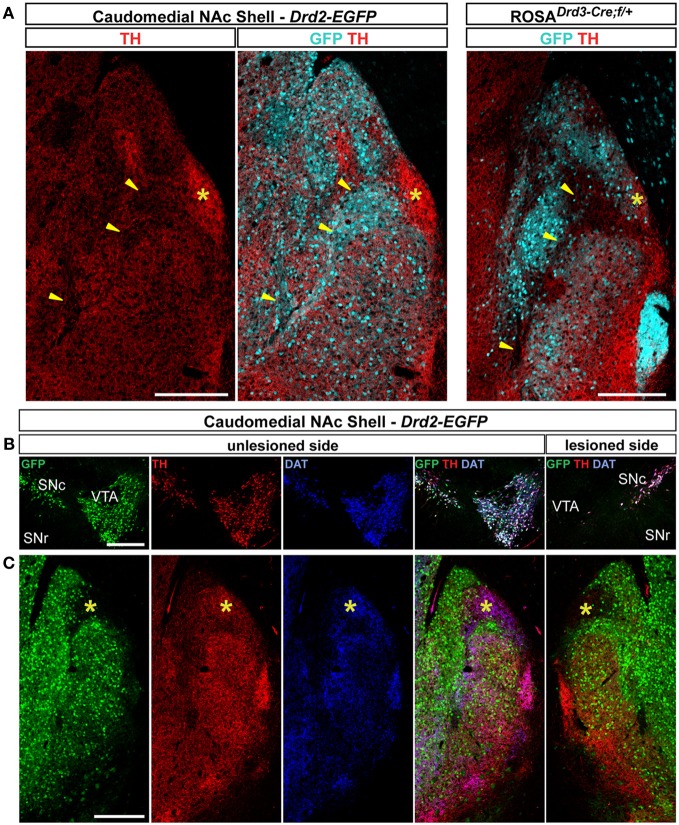
**Distribution patterns of tyrosine hydroxylase (TH) and dopamine transporter (DAT) in the caudomedial NAc shell. (A)** Distribution of TH (red) and GFP (cyan) fluorescence in the NAc of *Drd2-EGFP* and *Drd3-Cre/Rosa26:loxP* double transgenic mice. Yellow asterisk identifies the D2R-expressing MSNs-poor zone. Images are single confocal sections. Yellow arrowheads identify the bundle-shaped area. Scale bar: 250 μm. **(B)** Triple immunostaining for GFP (green), TH (red), and DAT (blue) allowed the identification of DA neurons on the unlesioned side of *Drd2-EGFP* mice. Note the lack of staining in the VTA on the lesioned side. Images are single confocal sections. Scale bar: 500 μm. **(C)** The same triple immunostaining performed at the caudomedial NAc shell level revealed the absence of DAT and a strong reduction of immunoreactive terminals in the D2R-expressing MSNs-poor zone (yellow asterisk). Images are single confocal sections. Scale bar: 250 μm.

The caudomedial part of the NAc shell exhibits inhomogeneous distribution patterns of various markers (Herkenham et al., 1984). We next assessed whether GFP distribution in *Drd2-EGFP*, *Adora2a-Cre*, and *Drd3-Cre* mice corresponded to cytoarchitecturally and cytochemically defined subterritories of the caudomedial part of the shell. Confirming previous studies, we observed that vesicular glutamate transporters 1 (VGluT1) and 2 (VGluT2) showed a complementary distribution in the NAc shell defining two well-separated neuronal circuits (Figures 6, 7) (Hartig et al., 2003). Thus, VGluT1 immunoreactivity was enriched in the bundle-shaped area where few D3R-expressing MSNs and an estimated high degree of D1R/D2R co-localization have been observed (Figure 6). In contrast, this subterritory was devoid of VGluT2-positive terminals and calretinin immunoreactivity (Figures 7A,B). On the other hand, in D2R-expressing MSNs-poor zone located in the upper part of the caudomedial shell, VGluT2-, and calretinin-positive fibers were dense whereas VGluT1 staining was weak (see the asterisks in Figures 6, 7). Interestingly, the pattern of TH immunoreactivity revealed a mosaic heterogeneity that resembled that of the VGluT2/calretinin distribution (Figure 8A). Therefore, the bundle-shaped area was identified as TH/DAT-poor area while the D2R-poor zone was stained by a dense plexus of TH/DAT fibers arising from the VTA (Figures 8B,C). It should be noted here that the remaining TH staining visible in the NAc shell following 6-OHDA-induced VTA lesion corresponded most likely to noradrenaline fibers arising from the locus coeruleus because they were devoid of DAT labeling (Figure 8C).

### Topographical and cell-type regulation of ERK phosphorylation in the NAc

Activated by a variety of therapeutic agents or drugs of abuse in physiological and pathological contexts, the ERK pathway has been proposed to play a critical role in the molecular mechanisms involved in dopamine-controlled striatal plasticity (Girault et al., 2007). Using *Drd2-EGFP* mice, we next analyzed the pattern of ERK phosphorylation following the administration of a D1R-like agonist (SKF81297, 5 mg/kg), a D2R-like agonist (quinpirole, 1 mg/kg), a non-selective dopamine receptor agonist (apomorphine, 3 mg/kg), and a D2R-like antagonist (raclopride, 0.3 mg/kg) in the core and in the various subterritories previously identified in the ventral and caudomedial part of the NAc shell.

#### NAc core

**Figure 9:**
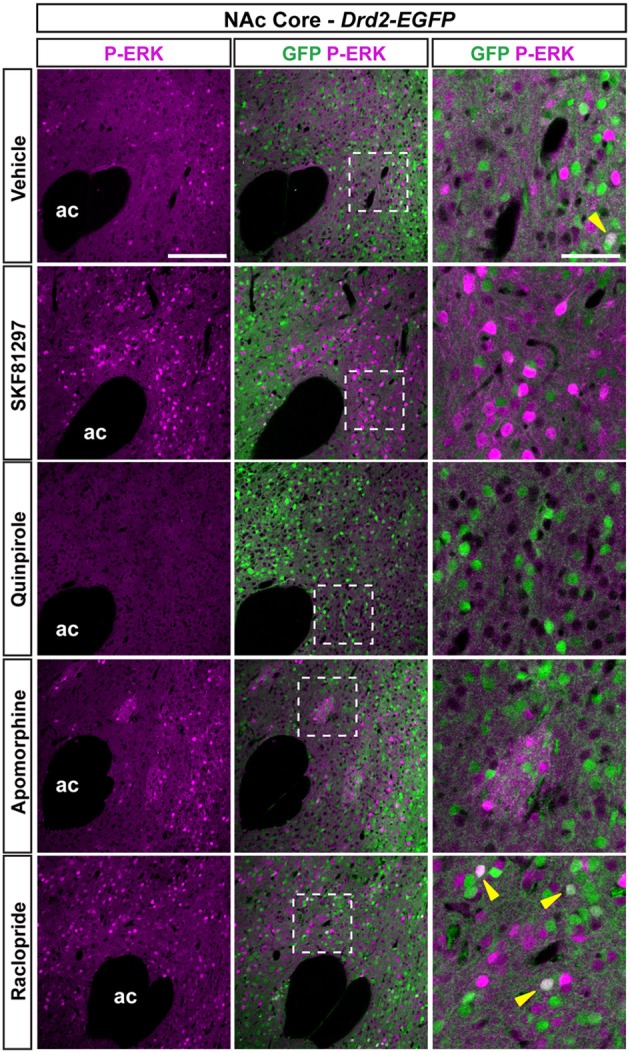
**Regulation of ERK phosphorylation in the NAc core.** Double immunofluorescence for P-ERK (magenta) and GFP (green) in the NAc Core of *Drd2-EGFP* mice treated with vehicle, SKF81297 (5 mg/kg), quinpirole (1 mg/kg), apomorphine (3 mg/kg), and raclopride (0.3 mg/kg). Scale bar: 200 μm. High magnification of the area delineated by the white dashed square. Yellow arrowheads identify D2R-expressing MSNs that contain P-ERK. Images are single confocal sections. Scale bar: 50 μm. Note that apomorphine-induced ERK phosphorylation in clusters that resemble the striosomal compartment.

**Figure 10:**
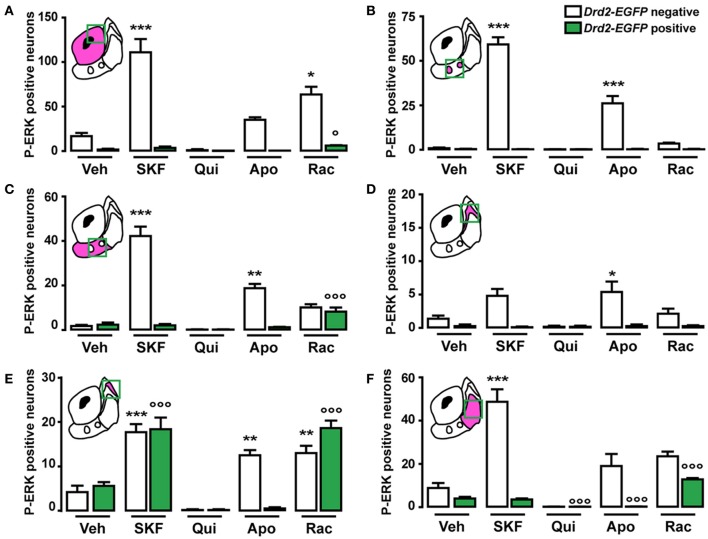
**Topographical and cell-type specific regulation of P-ERK by SKF81297, quinpirole, apomorphine, and raclopride in the NAc.** Quantification of P-ERK-positive cells among GFP-negative (white bars) and GFP-positive (green bars) neurons in the NAc core **(A)**, in the ventral NAc shell [**(B)** D2R-expressing MSNs-poor zone and **(C)** area surrounding the D2R-expressing MSNs-poor zone] and in the caudomedial NAc shell [**(D)** D2R-expressing MSNs-poor zone; **(E)** bundle-shaped area; and **(F)** cone] in *Drd2-EGFP* mice, 15 min after vehicle (Veh), SKF81297 (SKF), quinpirole (Qui), apomorphine (Apo), and raclopride (Rac) administration. Green squares in diagrams indicate the region analyzed. Data are number of cells per area (see Table A1) and are expressed as means ± SEM (3–5) and were analyzed using one-way ANOVA (see Table A3 for *F* values). Dunnett's test: ^*^p < 0.05, ^**^p < 0.01, ^***^p < 0.001 veh vs. drugs in *Drd2-EGFP* negative, °*p* < 0.05, °°°*p* < 0.001 veh vs. drugs in *Drd2-EGFP* positive.

As previously reported (Bertran-Gonzalez et al., 2008), vehicle-treated mice showed sparse P-ERK positive neurons that were only D2R-negative (Figures 9, 10A). Double fluorescence analysis of mice perfused 15 min after SKF81297 administration revealed that ERK phosphorylation occurred exclusively in D2R-negative neurons (Figures 9, 10A). In contrast, mice injected with quinpirole showed an inhibition of the basal ERK phosphorylation (Figures 9, 10A). Although apomorphine treatment did not significantly increase the total number of P-ERK-positive neurons, it induced areas of intense ERK phosphorylation in the neuropil (Figures 9, 10A). Finally, raclopride-treated mice showed a robust increase of ERK phosphorylation that occurred essentially in D2R-negative MSNs and to a smaller extent in D2R-expressing MSNs (Figures 9, 10A).

#### NAc ventral and caudomedial shell

**Figure 11:**
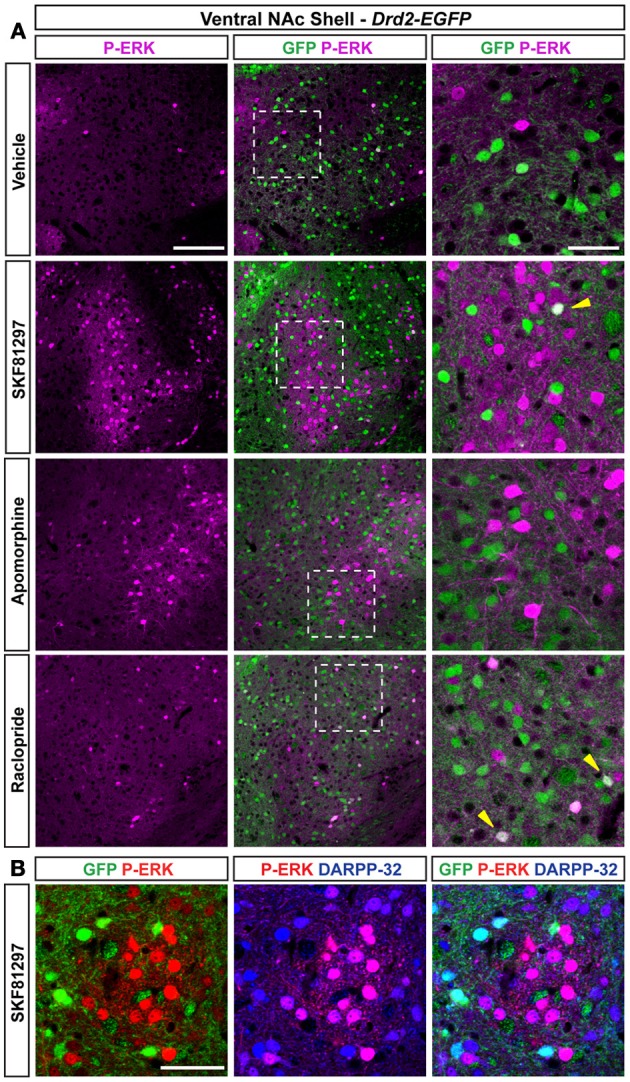
**Regulation of ERK phosphorylation in the ventral NAc shell. (A)** Double immunofluorescence for P-ERK (magenta) and GFP (green) in the NAc ventral shell of *Drd2-EGFP* mice treated with vehicle, SKF81297 (5 mg/kg), apomorphine (3 mg/kg), and raclopride (0.3 mg/kg). Scale bar: 100 μm. High magnification of the area delineated by the white dashed square. Yellow arrowheads indicate D2R-expressing MSNs that contain P-ERK. Images are single confocal sections. Scale bar: 50 μm. **(B)** P-ERK immunoreactivity (red) was detected together with DARPP-32 (blue) and GFP (green) immunoreactivities in the D2R-expressing MSNs-poor zone located in the ventral shell of *Drd2-EGFP* mice treated with SKF81297 (5 mg/kg) in a triple fluorescence analysis. Images are single confocal sections. Note that all P-ERK-positive cells co-localize with DARPP-32. Scale bar: 50 μm.

**Figure 12:**
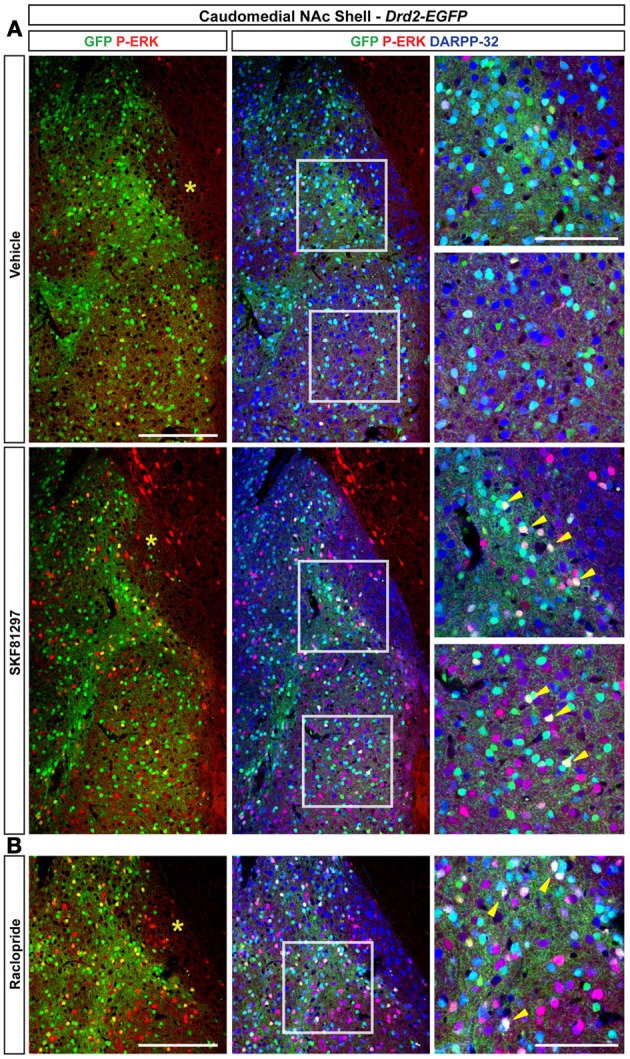
**Regulation of ERK phosphorylation in the caudomedial NAc shell. (A)** Triple immunofluorescence for P-ERK (red), GFP (green), and DARPP-32 (blue) in the caudomedial NAc shell of *Drd2-EGFP* mice treated with vehicle and SKF81297 (5 mg/kg) **(A)** and raclopride **(B)**. Images are single confocal sections. Scale bar: 200 μm. High magnification of the area delineated by the white squares. Scale bar: 100 μm. Yellow asterisk indicates the D2R-expressing MSNs-poor zone. Yellow arrowheads indicate P-ERK/GFP/DARPP-32 positive neurons.

Only few scattered P-ERK positive cells were detected in the ventral shell of vehicle-treated mice (Figures 10B,C, 11A). An inhomogeneous distribution was observed in the caudomedial part: basal ERK phosphorylation was observed in both D2R-positive and -negative MSNs in the bundle-shaped area enriched in D1R/D2R-co-expressing cells (Figures 10D–F, 12A), whereas P-ERK immunoreactivity was detected mostly in D1R-expressing neurons of the D2R-MSNs-poor zone and in the rest of the caudomedial shell. SKF81297 administration markedly increased the number of P-ERK-positive neurons in all subterritories of the ventral and the caudomedial shell with the exception of the D2R-expressing MSNs-poor zone (Figures 10B–F, 11A,B, and 12A). P-ERK immunoreactivity was detected only in D2R-negative cells, except in the bundle-shaped area where it was found in both cell types (Figure 10). Although less pronounced, a similar pattern of ERK activation was observed with apomorphine (Figures 10B–F, 11). The main difference concerned the cell-type specificity of ERK activation, which was restricted to D2R-negative cells in the bundle-shaped area (Figure 10E). As previously observed in the dorsal striatum (Gangarossa et al., 2012) and in the NAc core, quinpirole failed to induce ERK phosphorylation in any subterritory of the caudomedial and ventral shell analyzed (Figures 10B–F, 11). Finally, mice treated with raclopride also displayed a specific pattern of ERK activation: raclopride increased ERK phosphorylation in both D2R-negative and positive cells in the bundle-shaped area of the caudomedial shell whereas no change was found in the D2R-expressing MSNs-poor zones (Figures 10B,E, 11). P-ERK immunoreactivity was also slightly increased in the “cone” and ventral shell where a significant effect was observed in D2R-containing neurons (Figures 10B,F, 12B).

### Specific topographical and cell-type regulation of psychostimulant-induced ERK phosphorylation in the NAc

**Figure 13:**
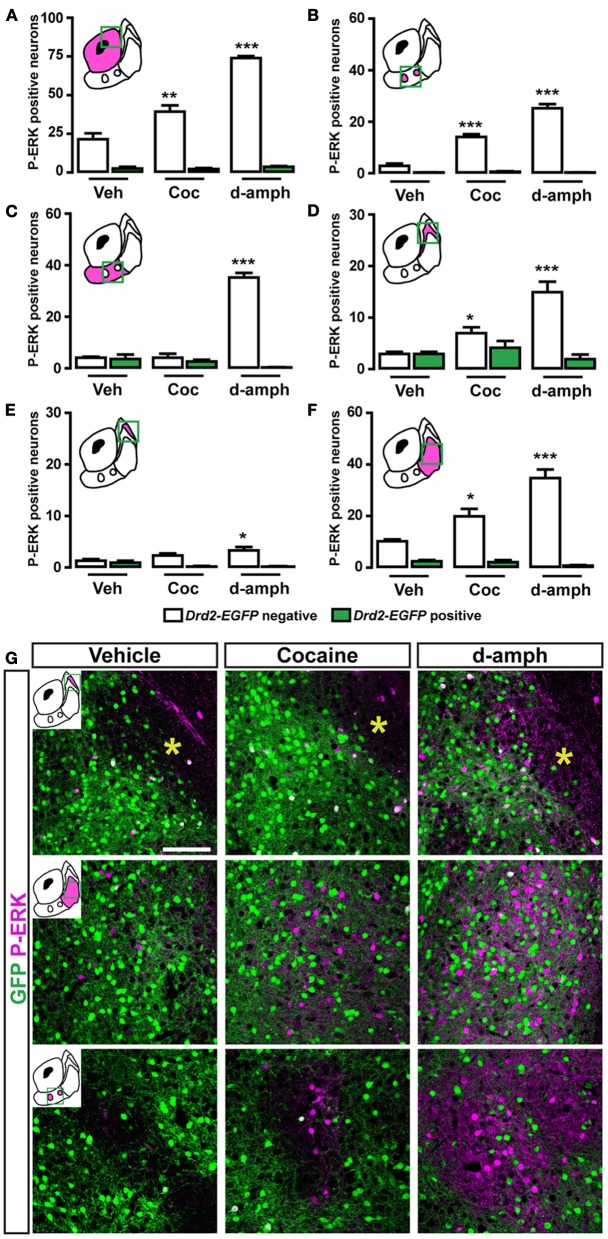
**Topographical and cell-type specific regulation of P-ERK by cocaine and d-amphetamine in the NAc. (A–F)** Quantification of P-ERK positive cells among GFP-negative (white bars) and GFP-positive (green bars) neurons in the NAc core **(A)**, in the ventral NAc shell [**(B)** D2R-expressing MSNs-poor zone and **(C)** area surrounding the D2R-expressing MSNs-poor zone] and in the caudomedial NAc shell [**(D)** bundle-shaped area; **(E)** D2R-expressing MSNs-poor zone; and **(F)** cone] in *Drd2-EGFP* mice, 15 min after vehicle (Veh), cocaine (Coc), and d-amphetamine (d-amph) administration. Note that cocaine- and d-amphetamine-induced ERK activation is restricted to D1R-expressing MSNs. Yellow asterisk indicates the D2R-expressing MSNs-poor zone. Green squares in diagrams indicate the region analyzed. Data are number of cells per area (see Table A2) and are expressed as means ± SEM (4–5) and were analyzed using one-way ANOVA (see Table A3 for *F* values). Dunnett's test: ^*^p < 0.05, ^**^p < 0.01, ^***^p < 0.001 veh vs. drugs in *Drd2-EGFP* negative. **(G)** Double immunofluorescence for P-ERK (magenta) and GFP (green) in various subterritories of the NAc shell in *Drd2-EGFP* mice treated with vehicle, cocaine (15 mg/kg) and d-amphetamine (10 mg/kg). Images are single confocal sections. Scale bar: 100 μm.

Acute cocaine treatment increases ERK phosphorylation in D1R-containing MSNs in the dorsal striatum and the NAc (Bertran-Gonzalez et al., 2008). Because of the inhomogeneous distribution of D1R- and D2R-expressing output neurons in the NAc, we examined the patterns of P-ERK-positive neurons taking into account the accumbal subterritories. A single injection of cocaine (15 mg/kg) or d-amphetamine (10 mg/kg) increased the number of P-ERK-positive neurons in D1R-containing MSNs in the NAc core (Figure 13A). In contrast, a more complex pattern of cocaine-induced ERK phosphorylation was observed within the NAc shell subterritories (Figures 13B–G). In the ventral part of the shell, cocaine increased ERK phosphorylation only in the zones lacking D2R-expressing MSNs while d-amphetamine also increased it in the surrounding area (Figures 13B,C,G). The analyses performed in the caudomedial part of the shell revealed that cocaine and d-amphetamine triggered almost similar patterns of ERK activation (Figures 13D–G). Thus, increased ERK phosphorylation restricted to D2R-negative MSNs was observed in the bundle-shaped area and the “cone” following administration of cocaine or d-amphetamine (Figures 13D,F,G). On the other hand, cocaine at this dose failed to activate ERK in the D2R-expressing MSNs-poor zone while d-amphetamine induced a weak increase (Figures 13E,G).

## Discussion

Recent advances in technologies for the identification of specific cell types, including BAC transgenic mice expressing fluorescent reporter or the Cre recombinase, allow a more comprehensive understanding of the involvement of D1R- and D2R-expressing MSNs in various physiological and pathological conditions. Although, potential caveats or difficulties in using such approaches have to be taken into account (i.e., incomplete and/or ectopic expression depending on the insertion site of the transgenes) (Gong and Yang, 2005), a careful identification and characterization of the mouse line that produces expression patterns matching that of the endogenous gene should avoid overstated conclusions. By using various BAC transgenic mice (*Drd1a-EGFP*, *Drd2-EGFP*, *Drd3-Cre*, and *Adora2-Cre*) combined with several immunohistochemical markers, we reveal a high level of heterogeneity of the NAc shell cellular organization. In the NAc core as in the adjacent dorsal striatum, D1R- and D2R-expressing MSNs appear to be randomly distributed. In contrast, in the ventral and the caudomedial part of the NAc shell, D1R- and D2R-expressing MSNs exhibit an inhomogeneous distribution. Identified patterns are closely associated with specific accumbal subterritories previously delineated by specific neurochemical markers. Figure A1 summarizes the main differences between the subterritories. Moreover, our results support the hypothesis that the heterogeneous composition of the NAc can be functionally important as illustrated by the distinct patterns of ERK activation triggered by pharmacological treatments.

### The bundle-shaped area

Generally seen as an integral part of the striatal complex, the identification of several anatomical features has led to propose that the NAc was an independent functional entity. Beyond the well-known NAc core and shell compartmentalization, selective markers and tract–tracing studies performed in rats allowed the identification of multiple accumbal shell subterritories (Zahm and Brog, 1992). Among them, cell clusters (Herkenham et al., 1984) or “corridors” (Seifert et al., 1998) have been identified in the caudomedial shell in the border region between the core and shell. Characterized by an enrichment of opioid receptors and low staining for acetylcholinesterase, substance P, and enkephalin (Herkenham et al., 1984; Voorn et al., 1989), the bundle-shaped area is also avoided by terminals originating from the ventral subiculum (Groenewegen et al., 1987), the infralimbic cortex (Berendse et al., 1992) and the paraventricular thalamic nucleus that are identified by VGluT2/calretinin immunoreactivity (Hartig et al., 2003). In addition, dopaminergic projections from the VTA also poorly innervate the bundle-shaped area as demonstrated by the weak density of dopamine, TH and DAT immunoreactive terminals (Voorn et al., 1989; Jansson et al., 1999, and present study). The paucity of these extrinsic afferent projections within the bundle-shaped area largely contributed to put forward the hypothesis that this accumbal subterritory would constitute a way-station favoring intrinsic information processing (Herkenham et al., 1984). However, the demonstration of a dense plexus of VGluT1 immunopositive fibers arising from the prelimbic cortex and the caudal parvicellular basal amygdaloid nucleus (Berendse et al., 1992; Wright and Groenewegen, 1996; Wright et al., 1996; Hartig et al., 2003) strongly supports the idea that MSNs located in the bundle-shaped area could also integrate and process specific cortical and subcortical information.

Our study clearly points out that the bundle-shaped area also displays several specific features regarding the distribution pattern of D1R- and D2R-expressing MSNs. Thus, this area can be identified by an enrichment of GFP immunofluorescence in *Drd2-EGFP* mice and a low number of D3R-containing MSNs (10% of all DARPP-32 immunoreactive cells). Interestingly, our estimated percent of D1R/D2R co-expression of 38% was roughly 2-fold higher than our previous evaluation in the whole NAc shell (Bertran-Gonzalez et al., 2008; Matamales et al., 2009). Although informative, these estimations should be taken with caution since numbers were obtained from different mice and calculations were based on the assumption that every MSN express either D1R or D2R or both in the case of the bundle-shaped area and either D1R or A2aR or both in the case of the D2R-expressing MSNs-poor zone in the caudomedial shell. Because recent studies suggest that MSNs co-expressing both receptors display unique signaling properties (Perreault et al., 2010, 2011), the bundle-shaped area would therefore represent an ideal anatomical substrate where D1R-D2R heteromers-dependent signaling could preferentially take place. In light of these observations, it is interesting to note that, because of the low TH and DAT expression, previous studies proposed that dopaminergic transmission in the bundle-shaped area results from a non-synaptic, volume transmission type of DA communication (Garris et al., 1994; Jansson et al., 1999). Further studies will be therefore necessary to determine whether DA-dependent signaling in the bundle-shaped area results in a prolonged action of DA as a consequence of a slow diffusion into the extracellular space following DA release from the rich surrounding DA networks.

### D2R-expressing MSNS-poor zones

Another level of compartmentalization of the NAc shell results from the existence of D2R-expressing MSNs-poor zones. Such areas located in the ventral shell have been identified. These zones contain neither A2aR nor D3R as demonstrated by the absence of GFP in the *Adora2-Cre* and *Drd3-Cre* mice, respectively. Therefore, the high number of DARPP-32-immunoreactive cells suggests that these D2R-expressing MSNs-poor zones are composed almost exclusively of D1R-contaning MSNs. It must be noted that the other histochemical markers used in previous or present studies did not allow the identification of this specific shell subterritory. Whether these clusters exhibit other particular features remains to be determined.

The second D2R-expressing MSNs-poor zone is located in the upper part of the caudomedial part of the shell. Identified as VGluT2-, calretinin-, TH/DAT-rich zone, this small area receives massive inputs from the paraventricular thalamic nucleus, the infralimbic cortex, and the VTA (Herkenham et al., 1984; Berendse et al., 1992; Groenewegen et al., 1999; Jansson et al., 1999; Hartig et al., 2003). Surprisingly, while D1R-expressing neurons represented 75% of DARPP-32-positive neurons, 93% of the MSNs in this area were GFP-positive in *Adora2a-Cre* mice suggesting (1) the existence of MSNs co-expressing D1R and A2aR and (2) the lack of co-localization between A2aR and D2R, which is normally observed in the dorsal striatum and others accumbal regions. These observations have important functional implications since the reciprocal antagonistic interactions between A2aR and D2R should not occur. This contrasts with the dorsal striatum, in which the co-expressed D2R and A2aR interact either directly, to form heteromers, or indirectly, at the level of adenylyl cyclase, to trigger the activation of specific signaling cascades (Ferre et al., 2011). Interestingly, half of the DARPP-32-containing neurons of this subterritory also express D3R suggesting that MSNs located in this area display a high degree of D1R, A2aR, and D3R co-expression. Whether this accumbal subterritory constitutes a “hot spot” where these receptors could interact and form functional D1R-D3R and A2aR-D3R heteromeric complexes will require further investigations (Torvinen et al., 2005; Fiorentini et al., 2008; Marcellino et al., 2008).

### Functional aspects of MSNs distribution in the NAc: impact on ERK activation

The pharmacological, physiological and pathological regulation of the ERK pathway in striatal and accumbal MSNs has been extensively studied (Girault et al., 2007; Santini et al., 2008; Gangarossa et al., 2012). Our present findings demonstrate the existence of a topographical and cell-type specific regulation of the ERK cascade signaling in the NAc in response to SKF81297, quinpirole, and apomorphine. As in the dorsal striatum, stimulation of D2R by quinpirole administration inhibited basal ERK phosphorylation in D1R-containing MSNs most likely as a result of DA release inhibition through the activation of D2 autoreceptors (Mercuri et al., 1997; Centonze et al., 2002; Gangarossa et al., 2012).

Following selective D1R stimulation, the most striking difference concerned the cell-type selectivity. Our data indicate that following SKF81297 administration ERK activation occurred in both D2R-positive and negative neurons in the bundle-shaped area. The most parsimonious explanation is that D2R-expressing MSNs in which ERK activation occurred could also contain D1R, a hypothesis supported by the high degree of D1R/D2R co-localization (38%) in this zone and indirectly by our results obtained with apomorphine. In that case, ERK phosphorylation occurred exclusively in D2R-negative MSNs suggesting that when both D1R and D2R are stimulated in the MSNs co-expressing them, ERK activation does not occur. In line with this hypothesis, a recent study showed that the co-activation of both receptors within the dopamine D1R-D2R heteromers by the selective D1R-D2R heteromer agonist SKF83959 failed to increase ERK phosphorylation in the NAc (Perreault et al., 2012).

In the dorsal striatum, the blockade of D2R by haloperidol or raclopride activates ERK selectively in D2R-expressing striatopallidal MSNs (Bertran-Gonzalez et al., 2008, 2009). Our study reveals different principles of regulation in the NAc. Thus, raclopride-induced ERK phosphorylation was observed exclusively in D1R-expressing MSNs in the NAc core and in both D2R and D1R-MSNs in the NAc shell. In the dorsal striatum, haloperidol-induced ERK activation in D2R-expressing MSNs involved A2aR (Bertran-Gonzalez et al., 2009). Given that raclopride produces a marked increase in extracellular adenosine in the NAc (Nagel and Hauber, 2004), it is tempting to speculate that similarly to the dorsal striatum, A2aR contributes to ERK activation in D2R-containing MSNs of the NAc shell following raclopride administration. On the other hand, the increase of ERK phosphorylation in D1R-expressing MSNs could result from the ability of raclopride to enhance DA release in the NAc core and shell (Aragona et al., 2008).

As previously reported cocaine- and d-amphetamine-induced ERK phosphorylation in the NAc was always restricted to D1R-expressing MSNs (Bertran-Gonzalez et al., 2008; Gerfen et al., 2008). Our study highlights an additional level of complexity since we show here that psychostimulants trigger specific patterns of ERK activation, which vary in the accumbal subterritories analyzed. In the ventral shell, contrasting with d-amphetamine, cocaine administration induced a small increase in the number of ERK-positive cells, which was restricted to the D2R-expressing MSNs-poor zone. A subterritory-specific ERK phosphorylation was also observed in the caudomedial NAc shell. Thus, ERK activation is restricted to the bundle-shaped area and surrounding zones but absent from the D2R-expressing MSNs-poor zone located in the upper part of the caudomedial shell. Several mechanisms could explain why psychostimulant drugs trigger compartmentalized patterns of ERK phosphorylation. First, the segregated activation of ERK could be directly linked to the various combinatorial expressions of D1R, D2R, A2aR, and D3R within the different accumbal subterritories. Thus, MSNs located in the D2R-expressing MSNs-poor zone, which display a high degree of D1R, A2aR, and D3R expression would have distinct signaling properties than MSNs co-expressing only D1R and D2R. Second, the inhomogeneous release of DA in the NAc shell following psychostimulants administration could be also an important factor that would drive this specific pattern of ERK phosphorylation (Aragona et al., 2008). Interestingly, recent studies revealed that in the NAc shell, different populations of DA neurons might release glutamate eliciting therefore excitatory postsynaptic responses in MSNs innervated by these DA neurons (Stuber et al., 2010; Tecuapetla et al., 2010). Whether those particular DA neurons also participate in cocaine-induced ERK regulation will require further investigations. Finally, given that the glutamatergic transmission largely contributes to psychostimulant-evoked ERK activation in the NAc (Valjent et al., 2000, 2005; Pascoli et al., 2011), it is tempting to speculate that specific inputs arising from distinct cortical, subcortical, and thalamic areas play also a critical role in the establishment of the compartmentalized ERK phosphorylation induced by cocaine and d-amphetamine.

In conclusion, we demonstrate that the inhomogeneous distribution of D2R-expressing MSNs allows defining subterritories in the NAc shell, which exhibit particular neurochemical and inputs-specific features. Combined with our *in vivo* functional signaling analysis, our study highlights the importance to precisely determine the neuronal populations in which signaling pathways are activated in order to better understand how they are regulated and what their corresponding functions are.

### Conflict of interest statement

The authors declare that the research was conducted in the absence of any commercial or financial relationships that could be construed as a potential conflict of interest.

## Appendix

Table A1 **Measurement of the surface areas of the NAc subregions analyzed in Figure 10**.

**Table:**

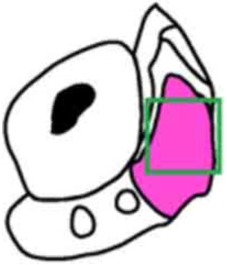

		**Veh**	**SKF**	**Qui**	**Apo**	**Rac**
	Area (mm^2^)	0.3119	0.3190	0.3363	0.3259	0.3273
	SEM (±)	0.0061	0.0074	0.0107	0.0102	0.0059
	*N*	4	5	3	4	4
	*F*-value	*F*_(4, 15)_ = 1.158; NS

**Table:**

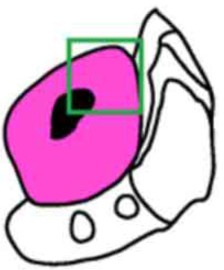

		**Veh**	**SKF**	**Qui**	**Apo**	**Rac**
	Area (mm^2^)	0.0618	0.0595	0.0497	0.0535	0.0541
	SEM (±)	0.0014	0.0057	0.0076	0.0013	0.0048
	*N*	4	5	3	4	4
	*F*-value	*F*_(4, 15)_ = 1.002; NS

**Table:**

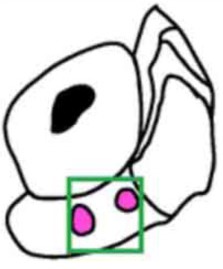

		**Veh**	**SKF**	**Qui**	**Apo**	**Rac**
	Area (mm^2^)	0.2403	0.2428	0.2747	0.2653	0.2588
	SEM (±)	0.0068	0.0080	0.0090	0.0080	0.0102
	*N*	4	5	3	4	4
	*F*-value	*F*_(4, 15)_ = 2.825; NS

**Table:**

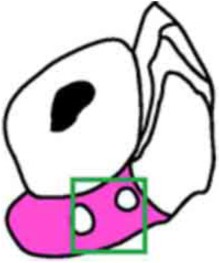

		**Veh**	**SKF**	**Qui**	**Apo**	**Rac**
	Area (mm^2^)	0.0509	0.0467	0.0385	0.0466	0.0458
	SEM (±)	0.0040	0.0064	0.0055	0.0016	0.0035
	*N*	4	5	3	4	4
	*F*-value	*F*_(4, 15)_ =0.7322; NS

**Table:**

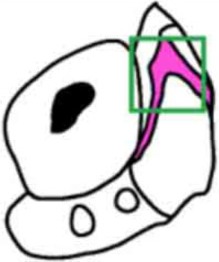

		**Veh**	**SKF**	**Qui**	**Apo**	**Rac**
	Area (mm^2^)	0.0373	0.0366	0.0372	0.0425	0.0363
	SEM (±)	0.0030	0.0010	0.0023	0.0016	0.0019
	*N*	4	5	3	4	4
	*F*-value	*F*_(4, 15)_ = 1.682; NS

**Table:**

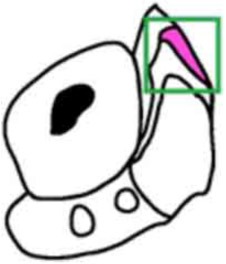

		**Veh**	**SKF**	**Qui**	**Apo**	**Rac**
	Area (mm^2^)	0.2021	0.2046	0.1949	0.2106	0.2034
	SEM (±)	0.0126	0.0067	0.0149	0.0018	0.0057
	*N*	4	5	3	4	4
	*F*-value	*F*_(4, 15)_ = 0.3593; NS				

Data are expressed as means ± SEM (n = 3–5) and were analyzed using one-way ANOVA followed by the Dunnett's post hoc test. NS: not significant.

Table A2 **Measurement of the surface areas of the NAc subregions analyzed in Figure 13**.

**Table:**

		**Veh**	**Coc**	**d-amph**
	Area (mm^2^)	0.3275	0.3088	0.3304
	SEM (±)	0.0078	0.0064	0.0050
	*N*	4	5	4
	*F*-value	*F*_(2, 10)_ = 3.429; NS

**Table:**

		**Veh**	**Coc**	**d-amph**
	Area (mm^2^)	0.0482	0.0489	0.0549
	SEM (±)	0.0041	0.0046	0.0049
	*N*	4	5	4
	*F*-value	*F*_(2, 10)_ = 0.6231; NS

**Table:**

		**Veh**	**Coc**	**d-amph**
	Area (mm^2^)	0.2836	0.2635	0.2716
	SEM (±)	0.0122	0.0080	0.0031
	*N*	4	5	4
	*F*-value	*F*_(2, 10)_ = 1.405; NS

**Table:**

		**Veh**	**Coc**	**d-amph**
	Area (mm^2^)	0.0464	0.0474	0.0519
	SEM (±)	0.0050	0.0041	0.0033
	*N*	4	5	4
	*F*-value	*F*_(2, 10)_ = 0.4495; NS

**Table:**

		**Veh**	**Coc**	**d-amph**
	Area (mm^2^)	0.0336	0.0318	0.0304
	SEM (±)	0.0040	0.0018	0.0004
	*N*	4	5	4
	*F*-value	*F*_(2, 10)_ = 0.4061; NS

**Table:**

		**Veh**	**Coc**	**d-amph**
	Area (mm^2^)	0.2016	0.2129	0.2107
	SEM (±)	0.0167	0.0056	0.0030
	*N*	4	5	4
	*F*-value	*F*_(2, 10)_ = 0.3741; NS		

Data are expressed as means ± SEM (n = 4–5) and were analyzed using one-way ANOVA followed by the Dunnett's post hoc test. NS: not significant.

**Table A3:** ***F*-values corresponding to the statistical analysis of the results presented in Figures 10 and 13**.

	**Drd2-negative cells**	**Drd2-positive cells**
Figure 10A	*F*_(4, 15)_ = 21.02, *p* < 0.001	*F*_(4, 15)_ = 6.30, *p* < 0.01
Figure 10B	*F*_(4, 15)_ = 80.37, *p* < 0.001	*F*_(4, 15)_ = 0.12, NS
Figure 10C	*F*_(4, 15)_ = 44.27, *p* < 0.001	*F*_(4, 15)_ = 9.74, *p* < 0.001
Figure 10D	*F*_(4, 15)_ = 4.78, *p* < 0.05	*F*_(4, 15)_ = 0.15, *p* NS
Figure 10E	*F*_(4, 15)_ = 20.48, *p* < 0.001	*F*_(4, 15)_ = 29.08, *p* < 0.001
Figure 10F	*F*_(4, 15)_ = 18.30, *p* < 0.001	*F*_(4, 15)_ = 99.33, *p* < 0.001
Figure 13A	*F*_(2, 10)_ = 55.35, *p* < 0.001	*F*_(2, 10)_ = 0.90, NS
Figure 13B	*F*_(2, 10)_ = 75.93, *p* < 0.001	*F*_(2, 10)_ = 1.57, NS
Figure 13C	*F*_(2, 10)_ = 151.20, *p* < 0.001	*F*_(2, 10)_ = 2.56, NS
Figure 13D	*F*_(2, 10)_ = 19.90, *p* < 0.001	*F*_(2, 10)_ = 1.20, NS
Figure 13E	*F*_(2, 10)_ = 4.30, *p* < 0.05	*F*_(2, 10)_ = 3.90, NS
Figure 13F	*F*_(2, 10)_ = 19.90, *p* < 0.001	*F*_(2, 10)_ = 2.32, NS

NS: not significant.
